# Ascertaining the Anatomical Parameters and Chemical Composition of *Luffa cylindrica* Cellulosic Fibers for Their Plausibility in Pulp and Paper Production

**DOI:** 10.3390/polym17192643

**Published:** 2025-09-30

**Authors:** Balasubramanian NagarajaGanesh, Balasubramanian Rekha, Manoharan Gopi Krishna, Syed Ibrahim Shaik Mohamed Ferozdheen

**Affiliations:** 1 Department of Mechanical Engineering, Anna University, Guindy, Chennai 600025, India; 2 Department of Physics, Manonmaniam Sundaranar University, Tirunelveli 627012, India; 3 Department of Mechanical Engineering, PSN College of Engineering and Technology, Melathediyoor, Palayamkottai, Tirunelveli 627152, India; 4 Department of Mechanical Engineering, Cape Institute of Technology, Levengipuram, Rajakrishnapuram, Tirunelveli 627114, India

**Keywords:** *Luffa cylindrica* fibers, cellulose, fiber morphology, derived indices, pulp and paper, sustainability, circular economy, paper industry

## Abstract

This research is mainly intended to assess the likelihood of producing pulp and paper from the cellulosic fibers of matured *Luffa cylindrica* fruit. The cellulose fibers were extracted and subjected to chemical composition studies and FTIR spectroscopic analysis. The chemical composition studies revealed that these fibers contain 82.4% holocellulose, 11.2% lignin, and 0.63% ash. Functional groups that represent the presence of the biopolymers were confirmed in the FTIR analysis. These fibers were observed through a light microscope, and important fiber parameters, such as the fiber diameter, fiber lumen, and cell wall thickness, were measured. Statistical analysis showed that the fiber dimensions follow a normal distribution. Based on the observed values, the derived indices that determine the fibers’ suitability to produce paper were calculated. The evaluated derived indices showed that the fibers possess a Runkel index of 59.67%, a slenderness ratio of 61.04%, a coefficient of rigidity of 63.7%, and a flexibility coefficient of 0.19. The Luce shape factor and Solids factor of the fibers were found to be 0.42 and 157.36 × 10^3^ μm^3^, respectively. This study proved that the morphology, derived indices, and chemical composition of the fibers are in par with other fiber sources that are used for pulp and paper production.

## 1. Introduction

Paper is a sheet of material that is usually made from materials such as wood, straw, bark, cellulose, rags, etc., which are transformed into a watery pulp of cellulose fibers. The word paper was derived from the Greek word pápūros, which was borrowed into Latin as papyrus, both referring to the *Cyperus papyrus* plant, whose pith was used by ancient Egyptians for writing. Paper is mainly used to prepare documents, paintings, wrappings, or to cover walls. Wood pulp is a raw material that is used for the production of paper that may be derived from both hardwood trees, such as birch, aspen, and eucalyptus, and from softwood trees such as pine, spruce, hemlock, and larch [1] (Dutt&Tyagi 2011). Paper has been used as a medium for storing valuable information for many years, and the need for it is indelible even in this digital age [2] (Gopal et al. 2019). Though the consumption of paper is on the rise, not all sectors are witnessing the same trend. For example, the development of modern computers, the internet, and mobile phones has caused a decline in the consumption of newsprint. On the other hand, of the total global consumption of 420 million tons of paper and paperboard in 2023, containerboard alone stood at 185 million tons and is expected to rise to 220 million tons by 2030 [3]. The containerboard type of paperboard is used in the manufacturing of corrugated boards. Sustainable consumption practices adopted during the purchasing of goods to reduce the use of single-use plastics, polythene bags, plastic straws, and takeaway cups have caused an increase in the use of packaging paper products. India is a growing economy, and the paper industry is highly clustered, with small-, medium-, and large-scale paper factories valued at INR 80,000, and it is growing annually at 8.2% per annum as per FY24 data. Of the 850 paper mills located geographically, only 550 are operational. The annual production capacity of the Indian paper industry is 25 million tons, and the count is increasing year by year. The per capita consumption of paper in India is between 15 and 16 kg, which is very far behind the global consumption of 57 kg. The Indian paper industry is in line with economic growth, and an increase in per capita consumption by 1 kg would lead to a demand of 1 million tons. Keeping this in view, the production volume is expected to reach 35 million tons by the fiscal year 2030, as per the report from the Indian Paper Manufacturers’ Association [4] https://ipmaindia.org/overview-2/ (Accessed on 10 September 2025). Apart from writing and printing, paper is used for wrapping, billing, copying, designing, education, and communication, making the need for it inevitable. The market capitalization of paper is forecasted to be USD 370 billion by 2027 [5] (Przybysz et al. 2018). The paper industry in India is facing challenges and is grappling with high raw material and import costs. The Directorate General of Commercial Intelligence and Statistics reports that India imported 1.145, 1.436, and 1.929 million metric tons of paper and paperboards during the fiscal years 2021–2022, 2022–2023, and 2023–2024, respectively [6]. This shows the increasing demand for paper and paper products year by year. The New Education Policy (NEP) 2023–2024 also positively influences the demand for paper due to the extended schooling system, reforms in higher education, and foundational literacy, making the market more attractive and optimistic. This shows the encouraging future prospects of this industry, which can be fuelled by increased paper production through the exploration of new resources for pulp and paper production. All these applications lead to the economic development of a country. The need and growth of paper usage can be witnessed from the increase in the production of pulpwood. This evinces that demand for pulp products will be increasing furthermore and hence alternate sources for producing pulp and paper products shall be vetted [7] (Anthonio&Antwi-Boasiako, 2017). Sufficient steps have to be taken to maintain a proper supply of pulp to the paper-producing industries so that they remain unaffected. This has spurred the motivation behind this study.

The basic raw materials used to produce paper in India are wood, bamboo, agriculture residue, and recycled fiber [8]. During the 1980s, the Indian paper industry used bamboo for 80% of paper production, and now the scenario has shifted from bamboo to recycled papers [6]. Of the total paper production, hardwood and bamboo contribute 21%, agro-waste contributes 8%, while the remaining 71% is through recycled fibers. To meet the increasing demand, India depends on imports. In 2024, India imported approximately 25 per cent of its wood pulp requirements from countries like Brazil and Indonesia, resulting in international supply chain risks and currency volatility [6]. Similar to this, many countries are unable to satisfy their paper needs, and hence, they cut trees and also try to mitigate paper imports by using alternative sources for producing paper and pulp. To accomplish this, non-wood fibers are also studied for pulp and paper production. Hence, remnants of agricultural produces like rice straws, wheat straws, sorghum, reeds, phragmites, cotton stalks, abaca, sugarcane bagasse, *Bambusastenostachya*, *Neosinocalamusaffinis,* and *Dendrocalamusstrictus* were studied by researchers to produce pulp [8,9,10,11] (Shakhes et al. 2011; Lukmandaru et al. 2016; Sadiku et al. 2016). Generally, different varieties of Eucalyptus trees, such as *Eucalyptus globules*, *Eucalyptus grandis*, *Eucalyptus camaldulesnsis*, *Eucalyptus regnans*, *Eucalyptus tereticornis,* etc., were found to be promising sources to produce paper and pulp [12,13,14,15] (Jorge, Qhilho and Pereira 2000; Ohshima, Yokota, Yoshizawa and Ona 2005; Sharma, Dutt, Upadhaya and Roy 2011; Ibrahim and Abdelazim 2015). All these varieties are fast-growing and are expected to deliver fiber woods within five to ten years after planting. Hence, they are mostly preferred for pulp and paper production. Studies on pulp and paper making show huge potential in lignocellulosic fibers, and more new species are being identified. In a recent review on the uses of sugar palm fibers, it was reported that these fibers can be used for making cigarette papers [16] (Sarwin Kumar et al. 2022). *Caesalpinia decapetela* fibers possess a holocellulose content of 78.14 wt% and lignin content of 18 wt%, making them a new raw material for pulp and paper [17] (Asrat et al. 2022). Discarded lignocellulosic fibers from *Cocos nucifera* fruit were explored recently for their prospects of pulp and paper production [18] (Ganesh et al. 2023). They were concluded to be a good source for pulp and paper production based on their fiber morphology and derived indices.

The prime requirement of fibrous and non-fibrous wood material to be used in making pulp and paper is dependent on its chemical composition and morphological features. Hence, these characteristics are studied before deploying a material to be used for producing paper and pulp. The morphological characteristics include fiber length, fiber diameter, lumen diameter, and cell wall thickness. The mechanical strength, efficiency, and quality of the pulp being produced depend on the chemical composition, fiber morphological characteristics, such as fiber length, fiber diameter, lumen width, wall thickness, etc. In addition, other traits, such as the microfibrillar angle, fiber surface roughness, or porosity, also influence pulp and paper properties [19] (Rekha 2022). These characteristics vary adversely between species and within the species on account of various external factors such as climatic conditions, soil type, age, and height of the plant, influencing the paper quality and the strength properties of the fibers [18] (NagarajaGanesh&Rekha 2019). Amongst the fiber parameters, fiber length influences the tear strength, and lengthy fibers are preferred in paper production. The strength of a paper, sheet density, and the pulp yield are controlled by the fiber wall thickness. Fibers with a large lumen width are called thin-walled fibers, and they ensure better beating and fiber flattening. Considering all these parameters, certain derived indices like the Runkel ratio, rigidity ratio, slenderness ratio, coefficient of flexibility, Luce’s shape factor, and the Solids factor were evaluated. These indices aid in adjudicating a fiber’s potential in making pulp of good quality. In addition to this, an investigation of the chemical contents, such as the holocellulose, lignin, and ash content in a fiber, is imperative. A high holocellulose content is considered favorable for pulp and paper production. Lignin content and ash content are undesirable and should be removed for pulp production, since they consume more chemicals, pulping time, and costs.

**Figure 1:**
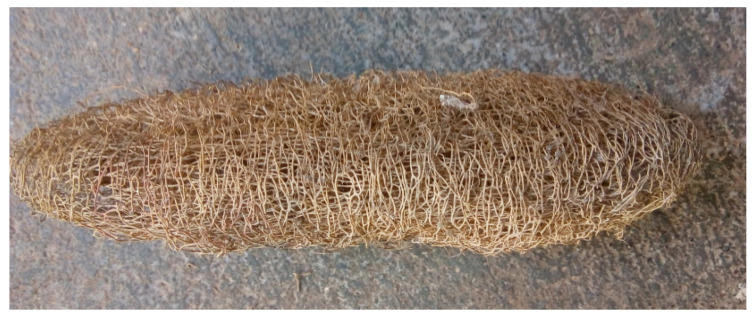
Completely ripened and matured *Luffa cylindrica* fruit.

In order to meet the increasing demand for pulp and paper products and to find new sources, the *Luffa cylindrica* plant was selected for this present study. The *Luffa cylindrica* plant is an edible climber that belongs to the Cucurbitaceae family and is found in South Asia. This plant produces a fruit that is edible when young. After maturity, this fruit develops into a strong fiber mesh and becomes inedible, as shown in Figure 1. This completely ripened and matured inedible fruit is left off unnoticed in the plant itself or used as a bath sponge. Fibers of this fruit were studied by many researchers for making many useful products like packaging items, composites, air filters, lintels, fences, decks, ceiling panels, cushions, etc. [20,21] (NagarajaGanesh and Muralikannan 2016; Tanobe et al. 2015). It also possesses some medicinal uses and is declared as an emerging cash crop. These fibers are stiff, exhibit less elongation, resulting in high tensile strength due to their low microfibrillar angle (NagarajaGanesh&Rekha 2020). Globally, China, Japan, Indonesia, Malaysia, Philippines, Hong Kong, Brazil, Caribbean Korea, Guatemala, Mexico, Ghana, Colombia, Venezuela, Nepal, and Central America are some of the top Luffa-producing countries. Young fruits are harvested for edible purposes, while matured fruit fibers are used as bath sponges, water purifiers, and in packaging applications. In the Southeastern United States, particularly in North Carolina and Florida, the yield of sponge gourds is more than 20,000 sponges per acre [22]. Spain, the major exporter of Luffa sponge gourds, exports Luffa fruit gourds worth USD 1.53 billion, and the USA imports sponge gourds worth USD 1.64 billion [23]. Regarding its availability in India, the cultivated area of *Luffa cylindrica* was approximately 24,800 acres, and the total production was 316,925 tons with an average yield of 39 tons per hectare as per the Government of India’s Indian Horticulture Database [24] (2019–2020). It was found that the fiber content in *Luffa cylindrica* fruit is 42.94 g per 100 g of fruit powder in immature fruits, while the dry and matured fruit is almost fibrous. This shows the potential and the demand for the *Luffa cylindrica* fibers.

The objective of this present work was to find the prospects of producing pulp and paper from inedible and discarded *Luffa cylindrica* fibers. These fibers were expected to meet the requirements for making pulp and paper that are similar to wood fibers based on their chemical composition and morphological features. Certain indices, called derived indices, should be evaluated from the fiber morphology perspective. Fibers that agree with these requirements are appropriate for pulping. If these requirements are met, then these fibers can be used for producing pulp and paper seamlessly. This can reduce the dependence and over-exploitation of commonly used fibers. Hence, in this study, chemical composition, Fourier transform infrared (FTIR) spectroscopic analysis, and morphological studies were conducted on the completely ripened *Luffa cylindrica* fibers to find their appropriateness for producing pulp and paper.

## 2. Materials and Methods

### 2.1. Collection of Materials

*Luffa cylindrica* fibers were taken from a matured *Luffa cylindrica* fruit in a plant at Pattam Village, Sivagangai District, Tamil Nadu State, India. The fruit was completely ripened and left unattached from the plant, making it fibrous. The outer cover of this inedible fruit was peeled off, and the inner core fibers were taken, cut, and cleaned with tap water continuously several times to eliminate dirt, mud, and other unwanted particles. Then, they were washed using distilled water and kept under sunlight for a week, and then used for further studies.

### 2.2. Determination of Chemical Composition

The moisture content in the extracted fibers was expelled by placing the fibers in an oven maintained at 105 °C for 4 h before further analysis. The holocellulose content was determined as per D1107-96 (2007) [18]. The lignin content was determined using the ASTM D1106-96 standards [18], prescribed as the standard method to determine the acid-insoluble lignin content present in the samples. A conical flask was taken, and 180 mL of distilled water was added to it. Then, sodium acetate, sodium chloride, and ethanolic acid of masses 8.6 g, 6.6 g, and 5.7 g, respectively, were added to it. The fiber sample was placed inside the conical flask and covered. It was kept under a fuming chamber for four hours, whose temperature was maintained at 60 °C. The solution changed its color, and the whitish sample was washed, filtered, and dried in an oven at a temperature of 105 °C for four hours and weighed. The ratio of the sample weight to the initial dry weight gives the wt% of holocellulose. A predetermined mass of the fiber was kept in a bath containing sulphuric acid. The temperature of the bath was maintained at 15 °C. After thorough mixing, it was transferred to a water bath maintained at 15 °C, followed by heating the contents for four hours. Later, the contents were filtered, dried, and weighed. The weight ratio of this dried lignin to the initial moisture-free mass shows the Klason lignin content. ASTM E1755-01 standard [18] was used to find the % of ash content present in these fibers. Two grams of the fiber sample were dried in an oven at 100–105 °C until its weight became constant. The carbon content present in the fibers was removed by heating the contents at a temperature of 550 °C using a muffle furnace. This was continued after alternate cooling and heating until a constant weight was obtained, and the weight was noted. The difference in weight of the sample before and after the heating shows the ash content present in the fibers.

### 2.3. Fourier Transform Infrared Spectroscopy

FTIR analysis of the *Luffa cylindrica* fibers taken from the ripened fruits was done using a Shimadzu (FTIR 8400S, Kyoto, Japan) spectroscope. Initially, the cleaned fibers were powdered and pressed using potassium bromide (KBr) powder, forming pellets. The spectrum was recorded in the wave number ranging from 500 to 4000 cm^−1^ at a 2 cm^−1^ resolution [21] (NagarajaGanesh&Muralikannan 2016). The analysis was conducted with a scan rate of 32 scans/min.

### 2.4. Determination of Fiber Dimensions

Fiber morphology is an important characteristic that tells the prospects of a fiber that is to be used for producing paper and pulp. Fiber length, fiber diameter, lumen diameter, and cell wall thickness are reported to control a fiber’s suitability in making paper [25] (Zobel and Van Buijtenen 1989). In this study, twenty samples of fibers were chosen and observed through a light microscope (Coslab, Ambala, India). Fiber morphology was studied in accordance with the standards specified by the International Association of Wood Anatomists (IAWA) (1989) [26]. Based on these dimensions of the fiber, namely the fiber diameter (FD), fiber length (FL), lumen width or lumen diameter (LW), and fiber wall thickness (FWT), the derived parameters discussed in the subsequent section were evaluated.

### 2.5. Statistical Testing

Statistical testing of the fibers was done with the Anderson–Darling test. This is a highly acknowledged test that is used to find any deviation of the values from normality and is basically used in research to find how well the data follows a distribution [26]. It involves the calculation of the Anderson–Darling indicator (AD) and the Adjusted Anderson–Darling indicator (AD*), applicable for small sample values. In this present study, this test was used to find whether the data were normally distributed or not. Generally, the Anderson–Darling test can be used for even 8 to 10 observations for certain distributions, unlike other tests. But sample sizes less than 8 and greater than 100 lack the power to detect deviations from normality and may reject the assumption of normality. The minimum advised sample size for this study was 20 [27] (Anderson and Darling 1954). Hence, as suggested, the sample size in this study was taken as 20.

Two hypotheses were stated and tested based on the *p*-value obtained in this method. The two hypotheses are the null hypothesis (H0) and the alternative hypothesis (H1).

**H0.**  *The data follow the normal distribution.*

**H1.**  *The data do not follow the normal distribution.*

The null hypothesis assumes that the data obtained are normally distributed, while the alternative hypothesis assumes that the data obtained are not normally distributed.

In this study, a *p*-value was determined for the Anderson–Darling test. This test is used to authenticate the significance of the data test. Based on the *p*-value, the hypothesis was tested, and a *p*-value less than or equal to 0.05 does not accept the null hypothesis, which shows that the data are not normally distributed. A *p*-value greater than 0.05 shows that the data obtained are normally distributed, accepting the null hypothesis. The significance level chosen for the test controls the *p*-value. This value is mostly chosen as 0.05 in the majority of the experiments, and this corresponds to a probability of 95%.

### 2.6. Determination of Derived Indices of Fibers

The commonly used derived indices that are used for finding a fiber’s potential in making pulp and paper are the Runkel ratio, coefficient of flexibility, slenderness ratio, rigidity ratio, Luce’s shape factor, and the Solids factor. All these indices were evaluated using the following Equations (1)–(6) [28,29,30,31] (Luce 1970; Malan &Gerischer 1987; Rana et al. 2009; Afrifah et al., 2020).

Runkel ratio = (2 × FWT/LW)(1)

Slenderness ratio = (FL/FD)(2)

Coefficient of flexibility = (LW/FD)(3)

Rigidity ratio = (FWT/FD)(4)

Luce’s shape factor = (FD^2^ − LW^2^)/(FD^2^ + LW^2^) (5)

Solids factor = (FD^2^ − LW^2^) × FL (6)

## 3. Results and Discussion

### 3.1. Chemical Composition

The chemical analysis test conducted on the *Luffa cylindrica* fibers shows that the holocellulose (Cellulose + hemicellulose) content in the *Luffa cylindrica* fibers was found as 82.4% by weight, with 63% cellulose and 19.4% hemicellulose. Fibers with a high holocellulose content (greater than 33%) are related to a high pulp yield and are mostly preferred [32] (Rowell 2012). Most of the plant species contain more than 70 wt% of holocellulose. This holocellulose content gives strength to the fibers because of the α-cellulose. The internal stress is reduced through hemicellulose. The holocellulose content of some fibers studied and found suitable for producing paper includes tobacco stalks (67.79%), canola stalks (73.6%), paulownia (75.74%), and reed (77.9%). The holocellulose content present in *Luffa cylindrica* fibers is more than these fibers and agrees well with that of G. wrayi (84.53%), G. levis (85.08%), and B. vulgaris (82.79%) [9] (Sadiku et al. 2016). The chemical constituents present in the *Luffa cylindrica* fibers are shown in Table 1. The cellulose content present in *Luffa cylindrica* fibers is 63% and is comparable with the cellulose content in *Rudbeckia laciniata*, *Fallopica bohemica*, *Rhusty phina,* and *Solidago canadensis,* which were found as efficient sources for paper making [33] (Kapun et al. 2022). The hemicellulose content in the fibers was found as 19.4 wt%, which is in agreement with the hemicellulose content of the eucalyptus species (18.88 wt%), evincing production of better quality paper [13,14] (Ohshima et al. 2005; Sharma et al. 2011). Hemicellulose, through hydrogen bonding, provides cross-linking of the cellulose microfibrils and is responsible for reducing the internal stress present in fibers. It increases paper strength, but too much hemicellulose is undesired for the pulping process. Lignin is another major constituent present in lignocellulosic fibers. It adds rigidity to the plant and increases bonding strength. But lignin creates difficulties in breaking the fiber bonds during the process of pulping. More lignin content consumes more chemicals and time during the process of pulping, which increases the pulping costs. Hence, fibers with a lignin content that is less than 30% are deemed fit for pulp and paper production. In this present study, the lignin content is 11.2%, which is agreeable and less than the lignin content present in pulp-producing fibers, such as tobacco stalks (18.9%), canola stalk (17.3%), paulownia (20.5%), and reed (18.7%) [8,34] (Enayati et al. 2009; Jalal shakes et al. 2011). Ash is another constituent in fibers, and it is the combustion residue left after heating the fibers to a temperature greater than 525 °C. The presence of metal salts like carbonates, phosphates, and oxalates of silicon, magnesium, manganese, and iron represents the ash content in fibers. High ash content is adverse to the pulping process as it causes recovery problems during cooking liquor by increasing the alkali consumption. It also affects pulp washing, beating, material handling, and bleaching operations. The lesser the ash content, the better the pulp quality. Ash content in the *Luffa cylindrica* fibers was found as 0.63%. This is less than the ash content in hardwood, softwood fibers, and many other fibers like cotton stalk (2.4%), reed (3.9%), and *Ananas comosus* (1.1%). All the constituents present in *Luffa cylindrica* fibers are within the admissible range specified for pulping, evincing that these fibers are suited for paper and pulp production [35,36] (Daud et al. 2014; Kiaet et al. 2014).

### 3.2. Fourier Transform Infrared Spectroscopy

**Figure 2:**
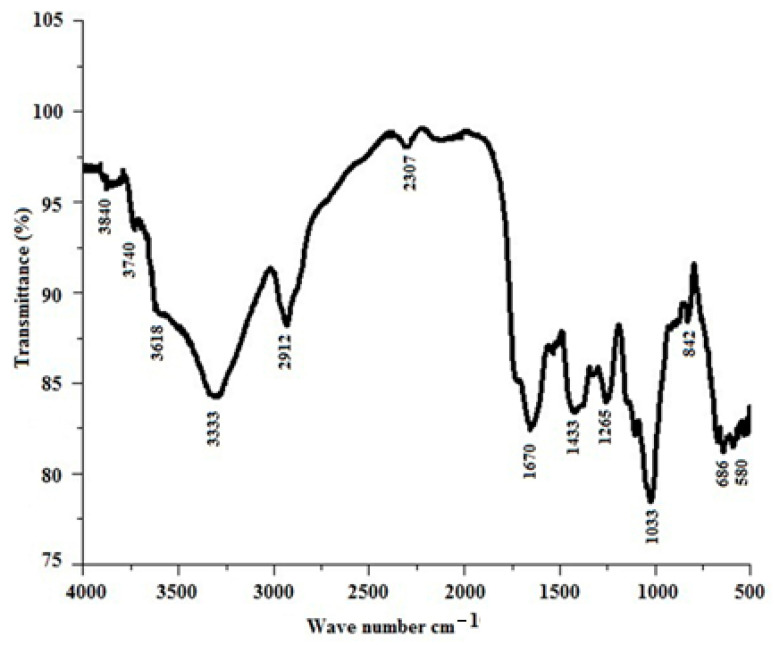
FTIR spectrum of the *Luffa cylindrica* fiber.

The FTIR spectrum of the *Luffa cylindrica* fiber is illustrated in Figure 2. The spectrum shows a broad nose peak at 3333 cm^−1^, which corresponds to the O-H stretching vibration of the polysaccharides present in the fibers [39]. Other peaks of less intensity are also seen at 3840, 3740, and 3618 cm^−1^. A sharp peak noticed at 2912 cm^−1^ is associated with the C-H asymmetric stretching, confirming the presence of fiber constituents, especially cellulose in the fibers. The tiny peak at 2307 cm^−1^ represents the O-H stretching of carboxylic acid. The sharp pointed peak at 1670 cm^−1^ is ascribed to the carboxylic groups (C=O) of lignin and hemicellulose in the fibers. The peak at 1433 cm^−1^ is associated with the CH_2_ bending vibrations, showing the presence of cellulose in them. The stretching vibration of the carbonyl (–C=O) bond is assigned to the peak at 1265 cm^−1,^ confirming the presence of lignin. The C-OH vibration that is assigned to hemicellulose is related to the sharp and pointed peak at 1033 cm^−1^. Small intensity peaks found at 842, 686, and 580 cm^−1^ are assigned to the symmetric ring-stretching mode of glycoside bonds, representing the presence of polysaccharides in the fibers. Thus, the peaks confirm the presence of fiber constituents from their functional groups. FTIR assignments of some commonly used lignocellulosic fibers used in papermaking are shown in Table 2.

### 3.3. Determination of Fiber Dimensions

#### 3.3.1. Fiber Length

Fiber length represents the count of bonding sites on the fiber that may be used to form a fiber network. The tear strength and folding endurance of a paper depend on the fiber length. In this present study, the fiber length of the *Luffa cylindrica* fibers was observed to lie in the range of 912–1108 μm. The mean fiber length was found as 995.6 ± 53.92 μm, which is in line with the fiber lengths of eucalyptus species, reported to lie between 670 and 1060 µm [1,42] (Dutt&Tyagi 2011; Ververis et al. 2004). The mean fiber length obtained is was slightly smaller than the non-wood fibers like tobacco stalks (1230 µm) canola stalks (1170 µm), corn stalk (1320 µm), and reeds (1390 µm), respectively, and was more than rice straw stalks (890 µm) and paulownia (820 µm) [34,43] (Enayati et al. 2009; Khakifirooz et al. 2012). Long fibers provide resistance against tearing of papers and are preferred, but too long fibers produce a less uniform sheet. Table 3 shows the dimensions of *Luffa cylindrica* fiber.

#### 3.3.2. Fiber Diameter

Fiber diameter is another morphological feature associated with pulp yield, and fibers with a small diameter provide flexibility and the required paper density. They form a high contact surface, and paper made with small-diameter fibers is strong with good tearing resistance. Flexible fibers offer good strength to fibers through more contact points, whereas fibers with a large diameter produce rough surface papers. The mean fiber diameter of bamboo, rice straw, reed, and pineapple fibers was reported as 15.1 μm, 13.5 μm, 14.8 μm, and 10 µm, respectively [14,37] (Sharma et al. 2011; Laftah&Rahman 2016). The mean diameter of *Luffa cylindrica* fibers was 16.31 ± 1.4 μm, similar to the aforementioned fibers, and hence papers produced using the *Luffa cylindrica* fibers will be dense and well-formed.

#### 3.3.3. Lumen Width

Fiber lumen width is a vital ultra-structural parameter that is related to the mechanical strength and thermal conductivity of fibers. Lumen is a tiny aperture through which necessary nutrients are inducted into the fiber and is also related to the pulping process. Beating of pulp is a process involved in pulping, and fibers with a large lumen width are susceptible to allowing more fluids into them, so that pulp yield would be high. A small lumen does not allow more fluids to pass through it during pulping, resulting in a low yield of pulp. Hence, fibers with a large lumen width are preferred. The lumen width of traditional sources of cellulose used for papermaking, namely *Eucalyptus grandis*, *Eucalyptus alba*, *tretecornis*, *torrelliana*, *europhyllia,* and *camaldulensis,* is 14.32 µm. 12.2 ± 3.2 µm, 9.8 ± 1.6 µm, 6.1 ±1.7 µm, 7.8 ± 2.4 µm, 6.1 ± 0.6 µm, 7.2 ± 0.9 µm, respectively. The lumen width of *Luffa cylindrica* fibers observed as 10.4 ± 1.29 μm in this present study is quite relative to the aforesaid eucalyptus species of fibers [1]. In addition, the lumen width values obtained in this study remain similar to the lumen width of canola stalk (12.5 μm), corn stalk (10.7 μm), *Cocos nucifera* (12.53 µm), and Jacitara palm (9.2 ± 3 µm) [14,18,44] (Sharma et al. 2011; Fonseca et al. 2013; NagarajaGanesh&Rekha 2023). Fibers with a smaller lumen diameter are suitable for making base papers for printed circuit boards, as they possess poor tensile strength.

#### 3.3.4. Fiber Cell Wall Thickness

The fiber cell wall is another anatomical parameter that should be considered for pulp production. Cell walls are initially thinner in nature during the young stage, and they become thicker towards maturity. It is used to find the age of a plant. Papers made from thick-walled fibers are coarse with less tensile strength and tearing resistance, with large void contents [42] (Ververis et al. 2004). Hence thin thin-walled fibers are preferred, and in this study, the fiber cell wall thickness of the fibers was found as 3.11 ± 0.76 μm. This value is smaller than many of the fibers, confirming that these fibers are suitable for manufacturing papers that are dense and smooth. The fiber wall thickness of reed and bamboo fibers was reported as 3.2 and 4.1 μm, respectively [14] (Sharma et al. 2011). Eucalyptus species, the long-established raw material for paper production, has cell wall thickness values lying between 3.29 and 3.86 µm concomitant with the values obtained in this study [1] (Dutt&Tyagi 2011).

### 3.4. Statistical Testing

In this present study, the Summation term S, Anderson–Darling indicator AD, and adjusted Anderson–Darling indicator AD* values for the fiber length, fiber diameter, lumen, and cell wall thickness of the observed data were calculated employing the Anderson–Darling technique [27] (Anderson and Darling 1954). The corresponding *p*-values were also calculated, and based on these values, the obtained data were tested for their deviation from normality, if any. Based on the above illustrations, considering the values observed in the experiment, the statistical calculations were made and shown in Table 3. The AD values of the fiber length, fiber diameter, fiber lumen, and cell wall thickness are shown. Since the number of samples was less, the corresponding AD* values were calculated. Then, based on the AD* values, the *p*-values of the fiber dimensions, such as the fiber length, fiber diameter, fiber lumen, and cell wall thickness, were calculated as shown. The *p*-values of fiber length, fiber diameter, fiber lumen, and cell wall thickness are reported in the last row of Table 3 under the respective row headings. It is obvious that the *p*-values obtained for the aforementioned fiber dimensions, such as the fiber length (0.871), fiber diameter (0.781), fiber lumen (0.101), and cell wall thickness (0.194) in the present research are all greater than 0.05. This shows that all the values pertaining to fiber dimensions follow a normal distribution.

**Figure 3:**
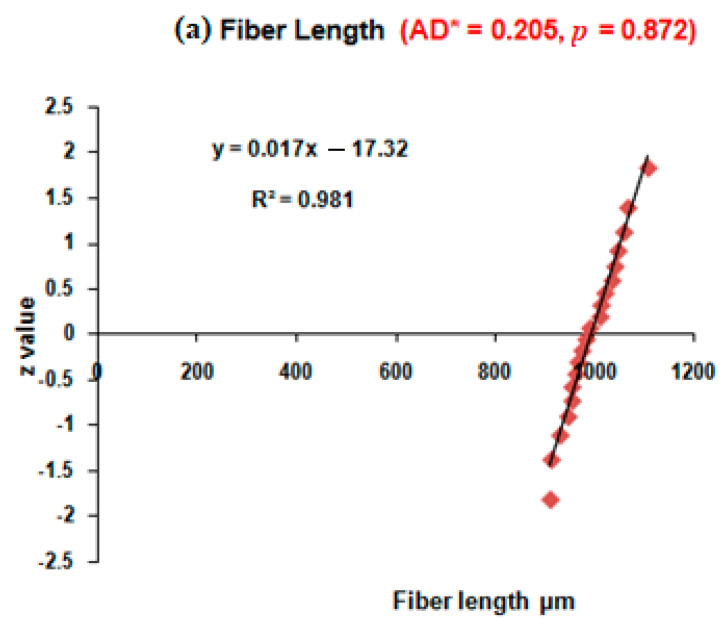

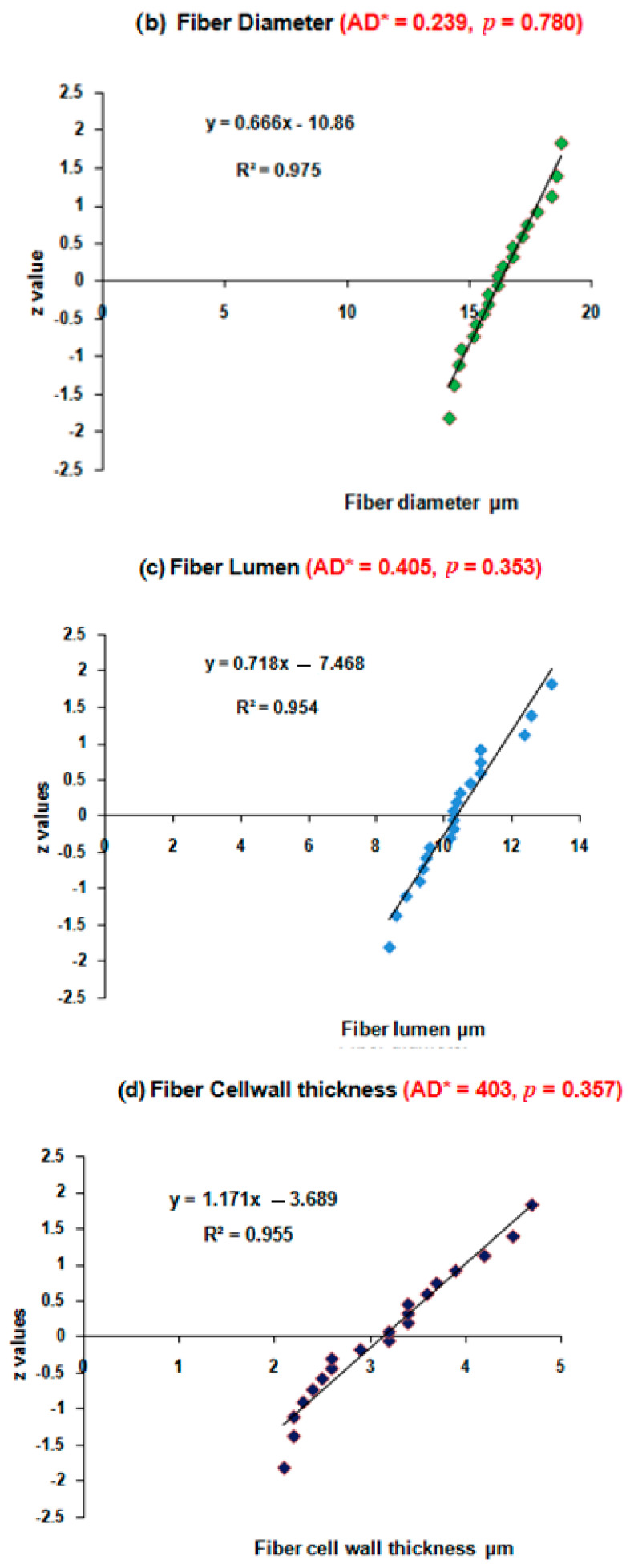
Normal probability plot of (**a**) fiber length, (**b**) fiber diameter, (**c**) fiber lumen, and (**d**) fiber cell wall thickness of the *Luffa cylindrica* fibers.

The Normal probability plots of the fiber length, fiber diameter, fiber lumen, and cell wall thickness of the *Luffa cylindrica* fibers are shown in Figure 3a, Figure 3b, Figure 3c and Figure 3d, respectively. As seen in these figures, the normal probability plot that provides the results of the AD–test and the *p*-values obtained in the research confirm that the fiber parameters obtained in this study fit a normal distribution.

### 3.5. Derived Indices of Fibers

Fiber’s capability to yield pulp and paper products can be verified using the derived indices. These derived indices help in identifying a fiber’s paper-producing potential from the calculated derived indices, namely the Runkel ratio, coefficient of flexibility, slenderness ratio, rigidity ratio, and Luce’s shape factor. These indices of the fibers were found and reported.

#### 3.5.1. Runkel Ratio

The Runkel ratio is computed as the ratio of the fiber cell wall thickness to its lumen width. It signifies the suitability of a fibrous material for pulp and paper production. Flexibility, bulkier nature, and stiffness of a paper were computed using this index [34,45] (Xu et al. 2006, Enayati et al. 2006). Thick-walled fibers produce bulky and poor-quality papers. Pulp produced using these fibers can be used as additives to replace hardwood fibers in small quantities to produce papers of various grades. Generally, fibers with a Runkel ratio less than 1 are preferred for producing quality papers, and the Runkel ratio of the *Luffa cylindrica* fibers is 0.5977, evincing that these fibers do not need more beating time for pulp production. Hence, time consumption is reduced and energy savings are achieved. This Runkel value is quite similar to the Runkel values of *Bambusa vulgaris, Setariaglauca,* and *Oxythenantera abyssinica* reported as 0.55, 0.57, and 0.60, respectively [14] (Sharma et al. 2015; [46] Boadu et al. 2020); Similar Runkel ratios were reported for the *Crotolarea pallida* (0.60) and *Sida cordifolia* (0.69) fibers, which were concluded to be suitable for pulp production.

#### 3.5.2. Slenderness Ratio

The slenderness ratio of a fiber is obtained by dividing the value of fiber length by fiber diameter. This factor shows the tearing property of paper. If the slenderness ratio of a fiber is greater than 33, then the fiber can be used for producing papers. But higher values are considered to produce high-quality paper. Slenderness values are used to classify the elastic nature of a fiber. According to [42] Ververis et al. (2004), if the slenderness ratio is greater than 75, the fibers are high elastic fibers. If the slenderness ratio is between 50 and 75, then the fibers are elastic in nature [42]. On the other hand, if the slenderness values of the fibers are in the range from 30 to 50, they are rigid fibers with very less slenderness values (<30). The slenderness ratio of *Cocos nucifera* (44.11), *Pinus kesiya* (57), and *Piccaabies* (53.96) fibers signifies that they are quite rigid [1,18] (NagarajaGanesh et al. 2023) and were used for pulping. In this present study, the slenderness value of the *Luffa cylindrica* fibers was found as 61.04, exemplifying that these fibers are elastic in nature. This value signifies the potential of these fibers in producing good-quality pulp. The slenderness ratio of *Alstonia boonei* was reported as 61% which is nearly equal to the slenderness value of the fibers used in the present study [47] (Afrifah&Adjei-Mensah 2021).

#### 3.5.3. Coefficient of Flexibility

The coefficient of flexibility of a fiber is usually expressed as a percentage and is deduced from the ratio of the lumen width to fiber diameter. This parameter determines the elasticity or rigidity nature of the fibers and controls the tensile strength, burst strength, and folding endurance of the papers [48] (Wangaard 1962). Coefficient of flexibility gives the bonding strength of an individual fiber and, by extension, the tensile strength and bursting properties [49] (Sangumbe et al., 2018). Based on the coefficient of flexibility, fibers are classified as highly rigid, rigid, elastic, and highly elastic with their respective coefficient of flexibility values less than 0.3, between 0.3 and 0.5, between 0.5 and 0.75, and greater than 0.75. The present study showed that the coefficient of flexibility of these fibers is 0.637, confirming the elastic nature of these fibers. Fibers of this type can be easily made into thin and strong sheets with a large surface area and nice bonding. Such papers are best suited for both writing and printing applications. This value is more than the flexibility coefficient of bamboo (0.41), less than *Pinus abies* (0.854) and *Pinus kesiya* (0.856) [1] (Dutt&Tyagi 2011). Similar types of values (0.61–0.69) were obtained for *Alstonia boonei* fibers [45] (Afrifah&Adjei-Mensah 2021). The flexibility coefficient values obtained for *Cocos nucifera* and *Chrysophyllum albidum* fibers were 0.59 and 0.57, respectively [50] (Samuel Ofosu et al. (2019). The obtained values show that these fibers can be used to produce good-quality printing papers.

#### 3.5.4. Rigidity Coefficient

The cell wall thickness of a fiber is used to determine the tearing resistance and the rigidity of a fiber. Dividing the cell wall thickness and the diameter of the fiber results in a parameter termed the rigidity coefficient. It is a measure that specifies a fiber’s pulp-making capability with respect to conformability and energy requirements. Rigidity values less than 0.5 are considered suitable for making pulp. To be precise, the rigidity coefficient values of soft and hardwood fibers should, respectively, lie in the range 0.13–0.2 and 0.15–0.35 for making high-strength papers. In the present case, the rigidity coefficient of *Luffa cylindrica* fibers is 0.19, indicating that these fibers require less energy and are highly conformable for producing less stiff, more flexible papers with substantial bonding strength. The rigidity values of *Solanum lycopersicum* (0.23) and *Capsicum annuum var. grossum* (0.24) support the values of the present research [51] (Sharma, Sharma, and Lama 2015). *Eucalyptus tereticornis* exhibited a high rigidity coefficient of 0.63, while the rigidity coefficient of *Bambusa* was 0.15.

#### 3.5.5. Luce’s Shape Factor

Luce’s shape factor is an important fiber index and is derived from the fiber diameter and lumen diameter, and it is directly related to a paper’s sheet density and shows the measure of resistance of the pulp to beating. It was evaluated using Equation (5), considering the fiber diameter and lumen width. Luce’s shape factor values less than 0.5 are considered good for paper and pulp making; however, eucalyptus species such as *Eucalyptus tereticornis*, *Eucalyptus camaldulensis,* and *Eucalyptus globules* were reported to possess values of 0.72, 0.37, and 0.42, respectively [12,13] (Ohshima et al. 2005; Pirralho 2014). All these species were reported to be used to make pulp and paper. It was reported that the Luce’s shape factor lies between 0.51 and 0.73 for *Spondia mombin* fibers [52] (Tembe 2021). The Luce’s shape factor of *Luffa cylindrica* fibers is 0.42. This value is less than 0.5 and agrees well with the values reported by researchers, making these fibers suitable for paper production. Other fibers like *Scoparia dulcis*, *Sida cordifolia,* and *Urena lobata*, that were considered for producing paper and pulp, exhibit a Luce’s shape factor value of 0.41 [1] (Dutt&Tyagi 2011).

#### 3.5.6. Solids Shape Factor

The Solids shape factor is another parameter that is similar to that of Luce’s shape factor and is inversely associated with the resistance of pulp to beating and the resistance of paper to bending. The fiber diameter, lumen width, and fiber length are the fiber parameters that influence the Solids shape factor of fibers. In this study, the Solids shape factor of *Luffa cylindrica* fibers is 157.3685 × 10^3^ μm^3^. This value is consistent with the values reported for *Alstonia boonei* and juvenile beech wood (140.380 × 10^3^ μm^3^) [47]. A Solids shape factor of 146 × 10^3^ μm^3^ was reported for the Hybrid Dwarf Yellow variety of *Cocos nucifera* fiber, which is well in agreement with the present research [31] (Afrifah, Osei, Ofosu 2020). The Solids shape factor of *Luffa cylindrica* fibers offers high resistance against bending and less resistance towards pulp beating, resulting in good-quality paper. Papers with good strength were obtained from fibers with a low Luce’s shape factor and Solids factor [14] (Sharma et al. 2018). The derived indices of some fibers recommended for paper making are shown in Table 4.

Derived indices, also called pulp quality indices, determine the aptness of a fiber to be used for pulping and paper production based on the fiber’s morphological traits. The values of the derived indices obtained in this present study are similar to the derived indices obtained for some common papermaking fibers found in the literature, as listed in Table 3.

Reliance on existing sources for papermaking may be a long route to meet the requirements. Also, deforestation to produce wood pulp causes problems like climatic change and environmental threats. Greater adoption of recycling of papers, adoption of 4.0 technologies, AI-enabled processes, IoT-driven technologies, and modernized equipment shall enhance the existing pulping systems and improve productivity. Other measures would be the exploration of new bio-alternatives from plants and agricultural byproducts for producing paper. These resources will not only enhance the livelihood of farmworkers but also increase the per capita income.

The chemical composition studies, FTIR analysis, and fiber morphological observations on the *Luffa cylindrica* fibers have shown that these fibers are capable of producing pulp and paper. The open and loose anatomical features of these fibers, along with their high cellulose, low lignin, and ash content, can aid in an effortless pulping process to produce good-quality bleachable pulp. The observed fiber dimensions, such as the fiber diameter, lumen diameter, cell wall thickness, and the derived indices, signify that these fibers do not require extensive refining to develop fibrillation. The fiber yield of this plant is 57.82%, agreeing with other pulp and papermaking coniferous plants that are reported as 50–60%, and is better than the *Rhus typhina, Robinia pseudoacacia,* and *Ailanthus altissima* fibers reported as 34–44% [33]. Further studies related to pulping, like cooking tests, pulp yields, and laboratory sheet properties, shall be conducted in the future. Intense pigmentation and objectionable odor are not present in these fibers, as claimed by the description in the patent [57]. The other advantage of the *Luffa cylindrica* plant is its easy adaptability to grow in all types of climatic and soil conditions. Recent studies conducted on these fibers unlock a multitude of applications. Based on the information collected through the internet, it seems that industrial production of cellulose from *Luffa cylindrica* fibers with subsequent processing is not found anywhere. *Luffa cylindrica* fibers are used in making bath sponges, matrix reinforcements, filters, packaging, water purifiers, etc. Thus cultivation of *Luffa cylindrica* fibers can be done on a large scale, and cellulose can be extracted from them for pulp and papermaking. Hence, these fibers may provide new vistas for the papermaking industry. The shortcoming associated with the *Luffa cylindrica* fibers is their coarseness and hard texture after maturity. These two characteristics and their torturous nature make it difficult to use directly. Nonetheless, this can be overcome by wall breaking and degumming the fibers using aqueous NaOH, dioxane, acetone, and pyridine [39,58,59]. Hence, it is ascertained that *Luffa cylindrica* cellulosic fibers are a viable source for producing pulp and paper seamlessly.

## 4. Conclusions

An initial study conducted on the fibers extracted from the matured and ripened *Luffa cylindrica* fruits satisfies the requirements for making good-quality pulp and paper similar to wood fibers. Adaptability to various climatic conditions, high fiber yields, and easy fibrillation of these fibers—simple cultivable measures—are some advantages of these fibers that can reduce the dependence and over-exploitation of other commonly used fibers for pulp and paper. In the present scenario, the challenges faced by the paper industry include higher import costs, paucity of raw material supply, dependence on wood pulp, a low recycling rate, supply chain problems, lower margins, and competition from foreign firms. Increasing demand for sustainable packing, environment friendly recycling measures, government’s educational initiatives, increased demand for paper and paper products, and a rise in per capita consumption are some of the existing opportunities that can be leveraged by finding an alternative biodegradable raw material. In this direction, this study elicits a new opportunity for the *Luffa cylindrica* fibers. Policy makers shall focus on innovating modern recycling techniques, provide incentives to farmers who cultivate raw materials for pulping, and encourage startup firms to develop new methods and materials for paper production. These interventions can help in achieving the UN’s Sustainable Development Goals (SDGs).

## Figures and Tables

**Table 1:** Chemical composition of some fibers used for pulp and paper making.

Fiber Name	Holocellulose	Lignin	Ash Content	References
	wt%	wt%	wt%	
*Eucalyptus grandis*	59.8	29.6	2.87	[1]
*Eucalyptus alba*	60.3	27.9	0.36	[1]
*Eucalyptus europhyllia*	64.2	26.5	0.98	[1]
Reed	77.9	18.7	3.9	[8]
Paulownia	75.4	20.5	0.21	[8]
Canola stalk	73.6	17.3	8.2	[8]
*Bamboosa vulgaris*	79.01	40.41	29.2	[9]
*Eucalyptus pellita*	71.07	25.5	-	[11]
*E. tereticornis*	66.5	28.2	1.12	[1]
*Ananas comosus*	82	5–12%	1.1	[37]
Softwood	60–80	21–37	<1	[35]
Hardwood	71–89	14–34	<1	[36]
Rice straw	70.85	17.2	16.6	[38]
Tobacco stalks	67.79	18.9	6.86	[8]
*Luffa cylindrica*	82.4	11.2	0.63	Current work

**Table 2:** FTIR band assignments of lignocellulosic fibers used in papermaking.

Wave Numbers (cm^−1^)						
Eucalyptus Wood	Bagasse	Unbleached Wood Pulp	Bleached Wood Pulp	Flax Straw	*Luffa cylindrica*	Assignment
3338	3338	3339	3337	3500–2900	3333	O-H stretching vibration
2892	2917	2898	2896	2911	2912	C-H stretching
1424	1423	1433	1426	1420–1430	1433	C-H bending
1231	1240	1249	1204	1230	1265	Out of plane C-OH
1031	1033	1031	1055	1066	1033	C-OH bending
896	896	897	897	900	842	Glucosidic ring stretching
[40]	[40]	[40]	[40]	[41]	This work	References

**Table 3:** Dimensions of *Luffa cylindrica* fiber.

S.No	Fiber Length	Fiber Diameter	Lumen Diameter	Cell Wall Thickness
	µm	μm	μm	µm
1	956	15.2	9.3	2.9
2	984	16.2	9.5	3.4
3	964	14.7	10.3	2.3
4	1022	16.4	12.6	2.2
5	1014	16.2	10.2	3.2
6	1036	17.4	10.3	3.6
7	1012	18.6	14.2	2.4
8	956	15.3	11.1	2.3
9	976	15.8	8.9	3.4
10	932	16.8	10.8	3.2
11	912	14.6	11.1	2.1
12	948	14.4	9.4	2.5
13	914	14.2	10.4	2.2
14	1042	18.8	10.5	4.2
15	968	15.8	9.6	3.3
16	992	15.6	8.6	3.6
17	1068	17.8	12.4	2.6
18	1060	16.8	8.4	4.5
19	1048	17.2	10	3.7
20	1108	18.4	10.2	4.4
Mean	995.6	16.31	10.39	3.1
Min	912	14.2	8.4	2.1
Max	1108	18.8	14.2	4.5
Range	196	4.6	5.8	2.4
STD	53.92	1.40	1.41	0.76
CV%	5.42	8.58	13.57	24.52
N	20	20	20	20
S	−403.940	−404.578	−412.037	−409.828
AD	0.197	0.229	0.602	0.491
AD*	0.205	0.239	0.628	0.513
*p*-Value	0.872	0.781	0.102	0.194

STD—standard deviation; CV—coefficient of variance; N—sample size; S—summation term; AD—Anderson–Darling indicator; AD*—adjusted Anderson–Darling indicator.

**Table 4:** Derived indices of some common fibers used for paper making.

Fiber Name	Runkel Ratio	Slenderness Ratio%	Coefficient of Flexibility	Rigidity Ratio	Luce Shape Factor	Solids Factor 10^3^ μm^3^	References
*Biden spilosa*	0.46	47.33	69.01	-	0.33	-	[1]
*Eupatorium odoratum*	0.52	42.3	65.81	-	0.50	-	[1]
Tobacco stalk	1.16	50.59	63.26	-	-	-	[8]
*Setaria glauca*	0.57	173.15	65.57	0.35	0.4	0.86	[14]
*Solanum torvum*	0.25	24.62	80.30	-	0.19	-	[14]
*Pinus kesiya*	0.22	49.04	81.74	-	0.19	-	[14]
*Cocos nucifera*	0.67	44.11	0.59	0.19	0.486	278.53	[18]
*Beema bamboo*	0.69–0.8	78.38–101.48	56.32–60.39	-	-	-	[46]
*Oxythenantera abyssinica*	0.6–0.76	82.37–93.92	59.75–60.66	-	-	-	[46]
*Chrysophyllum albidum*	0.55	46.9	0.64	0.177	0.41	346	[50]
*Saccharum officinarum*	2.479	69.77	29.12	0.722	84	634	[53]
*Eucalyptus tereticornis*	1.047	39.07	39.73	0.416	0.727	256	[53]
*Leucaena leucocephala*	0.59	-	0.63	-	0.41	-	[54]
*Bambusa. vulgaris*	2.82	88.96 ± 0.8	27	-	-	-	[55]
*B. longispiculata*	3.28	76.08 ± 3.5	24	-	-	-	[55]
*Dendro calamus*	3.39	91.17 ± 1.34	23.8	-	-	-	[55]
*Thyrsocalamus liang*	4.21	84.42 ± 0.55	19.8	-	-	-	[55]
*Debdro asper*	5.43	116.14	16	-	-	-	[55]
*Acacia mangium*	0.45	56.5	0.69	0.15	0.36	156	[56]
*Luffa cylindrica*	0.5977	61.04	0.637	0.19	0.42	157.36	Current work

## Data Availability

Data are contained within the article.
